# Experiments and Observations on the Different Modes in Which Death Is Produced by Certain Vegetable Poisons

**Published:** 1811-08

**Authors:** B. C. Brodie


### *Experiments and Observations on the different Modes in which Death is produced by certain vegetable Poisons.*

*By* Mr. B. C. Brodie, F. R. S.

(Phil. Trans.)

*Read before the Royal Society, February* 21, 1811.

I. THE following experiments were instituted with a view to ascertain, in what manner certain substances act on the animal system, so as to occasion death, independently of mechanical injury. I was led to the inquiry, from the subject of it appearing to be of considerable interest and importance, and from a hope, that, in the present improved state of physiological knowledge, we might be enabled to arrive at some more satisfactory conclusions, than had been deduced from any former observations.

The substances which act as poisons when applied to the animal body are very numerous. In the experiments, which I have hitherto made, I have employed vegetable poisons only. Of these I have selected such as are very active and certain in producing their effects, believing that, on this account, the exact nature of those effects would be more readily ascertained. The principal objects which I have kept in view have been to determine, on which of the vital organs the poison employed exercises its primary influence, and through what medium that organ becomes affected. I have also endeavoured to ascertain by what means the fatal consequences of some poisons may be prevented. With some of the conclusions, which I have ventured to draw, so far as I know, we were not before acquainted; and others of them, though not entirely new, had not been previously established by satisfactory experiments.

I shall relate first those experiments, in which poisons were applied internally, that is, to the mucous membranes of the tongue or alimentary canal, and afterwards those, in which poisons were applied to wounded surfaces.

### II. *Experiments with Poisons applied to the Tongue or alimentary Canal.*

### *Experiments with Alcohol.*

When spirits are taken into the stomach, in a certain quantity, they produce that kind of delirium which constitutes intoxication: when taken in a larger quantity, it is well known that they destroy life altogether, and that in the course course of a very short space of time. Intoxication is a derangement of the functions of the mind, and, as these are in some way connected with those of the brain, it seems probable, that it is by acting on this organ that spirits when taken into the stomach occasion death. In order to ascertain how far this conclusion is just, I made the following experiments.*

*Experiment* 1. I poured two drams of proof spirits down the œsophagus of a cat. Instantly he struggled violently; then lay on one side, perfectly motionless and insensible; the breathing was laboured and stertorous, and the pulsations of the heart were very frequent. He continued in this state for seven or eight minutes; then began to recover; the respiration became easier, and presently he stood up, and was able to walk.

*Exp.* 2. I injected an ounce and a half of proof spirits into the stomach of a large full-grown rabbit, by means of an elastic gum tube passed down the œsophagus. The same symptoms took place as in the last experiment; but the animal did not begin to recover from the state of insensibility, until forty minutes had elapsed from the time of the injection.

*Exp.* 3. Seven drams of proof spirits were injected into the stomach of a younger rabbit. Two minutes afterwards, he evidently was affected by the spirits, and in three minutes more he lay on one side motionless and insensible. The pupils of the eyes were perfectly dilated; there were occasional slight convulsive motions of the extremities; the respiration was laborious, it was gradually performed at longer and longer intervals, and at the end of an hour and fifteen minutes had entirely ceased. Two minutes after the animal was apparently dead, I opened into the thorax, and found the heart acting with moderate force and frequency, circulating dark coloured blood. I introduced a tube into the trachea, and produced artificial respiration by inflating the lungs, and found that by these means the action of the heart might be kept up to the natural standard, as in an animal from whom the head is removed.

*Exp.* 4. I injected into the stomach of a rabbit two ounces of proof spirits. The injection was scarcely completed, when the animal became perfectly insensible. Precisely the same symptoms took place as in the last experiment, and at the

* I am indebted to Dr. E. N. Bancroft for his assistance in many of the experiments which I am about to detail. Mr. W. Brande lent me his assistance in the greater part of those which were made. I have been further assisted by Mr. Broughton, Mr. R. Rawlins, and Mr. R. Gatcombe, and by several other gentlemen.

end end of twenty-seven minutes, from the time of the injection, the rabbit was apparently dead; but on examining the thorax the heart was found still acting, as in the last experiment.

It has been shewn by M. Bichat, and the observation has been confirmed by some experiments, which I have lately had the honour of communicating to this learned Society, that the brain is not directly necessary to the action of the heart, and that, when the functions of the brain are destroyed, the heart continues to contract for some time afterwards, and then ceases only in consequence of the suspension of respiration, which is under the influence of the brain.

It would appear, from the experiments, which I have just detailed, that the symptoms produced by a large quantity of spirits taken into the stomach, arise entirely from disturbance of the functions of the brain. The complete insensibility to external impressions; the dilatation of the pupils of the eyes; and the loss of motion, indicate that the functions of this organ are suspended; respiration, which is under its influence, is ill performed, and at last altogether ceases; while the heart, to the action of which the brain is not directly necessary, continues to contract, circulating dark coloured blood for some time afterwards.

There is a striking analogy between the symptoms arising from spirits taken internally, and those produced by injuries of the brain.

Concussion of the brain, which may be considered as the slightest degree of injury, occasions a state of mind resembling intoxication, and the resemblance in some instances is so complete, that the most accurate observer cannot form a diagnosis, except from the history of the case. Pressure on the brain, which is a more severe injury than concussion, produces loss of motion, insensibility, dilatation of the pupils; respiration becomes laboured and stertorous, is performed at long intervals, and at last altogether ceases, and the patient dies.

It forms an interesting matter of inquiry, whether spirits when taken into the stomach produce their effects on the brain, by being absorbed into the circulation, or in consequence of the sympathy that exists between these organs by means of the nerves. The following circumstances lead me to conclude that they act in the last of these two ways.

1. In experiments where animals have been killed by the injection of spirits into the stomach, I have found this organ to bear the marks of great inflammation, but never found any preternatural appearances whatever in the brain. 2. The effects of spirits taken into the stomach in the last experiment were so instantaneous, that it appears impossible that ab-

 sorption sorption should have taken place before they were produced. 3. A person who is intoxicated, frequently becomes suddenly sober after vomiting. 4. In the experiments, which I have just related, I mixed tincture of rhubarb with the spirits, knowing from the experiments of Mr. Home and Mr. William Brande, that this, when absorbed into the circulation, was readily separated from the blood by the kidneys, and that very small quantities might be detected in the urine by the addition of potash; but, though I never failed to find urine in the bladder, I never detected rhubarb in it.

The including the termination of the thoracic duct in a ligature does not prevent spirits, when taken into the stomach, from producing their usual effects on the nervous system, but subsequent observations, which Mr. Home has already communicated to this Society, have shewn that no conclusion can be drawn from this experiment.

That a poison may affect a distant organ, through the medium of the nerves, without entering the circulation, is proved by the well-known circumstance of solution of the extract of *Belladonna*, when applied to the tunica conjunctiva of the eye, occasioning dilatation of the pupil of the same eye, though no other part of the system is affected.

It has been formerly supposed by Dr. Mead and other physiologists, that a poison may produce death by acting on the extremities of the nerves of the stomach and intestines, without being absorbed into the circulation. That it should by these means be capable of affecting the brain is not to be wondered at, when we consider the numerous and various sympathies between this organ and the alimentary canal, evidently independent of any other communication than the nerves.

### *Experiments with the Essential Oil of Bitter Almonds.**

*Experiment* 5. One drop of the essential oil of bitter almonds was applied to the tongue of a young cat. She was instantly seized with violent convulsions; then lay on one side motionless, insensible, breathing in a hurried manner; the respirations became laboured, took place at longer and longer intervals, and at the end of five minutes, from the application of the poison, had entirely ceased, and the animal was apparently dead; but on opening the thorax, the heart

* The essential oil of bitter almonds does not appear to differ from the essential oil of laurel. I was furnished with a quantity of it, first by my friend Mr William Brande, and afterwards by Mr. Cooke of Southampton-street.

was was found acting regularly eighty times in a minute, circulating dark coloured blood, and it continued to act for six or seven minutes afterwards.

*Exp.* 6. I injected into the rectum of a cat half an ounce of water, with two drops of the essential oil. In two minutes afterwards, he was affected with symptoms similar to those which occurred in the last experiment, and at the end of five minutes, from the injection of the poison, he was apparently dead. Two minutes after apparent death, the heart was found acting eighty times in a minute. On dissection, no preternatural appearances were found either in the internal membrane of the rectum, or the brain.

The symptoms produced by this poison, and the circumstance of the heart continuing to contract after apparent death, lead to the conclusion that it occasions death by disturbing the functions of the brain.

While engaged in these last experiments, I dipped the blunt end of a probe into the essential oil, and applied it to my tongue, meaning to taste it, and having no suspicion that so small a quantity could produce any of its specific effects on the nervous system; but scarcely had I applied it, when I experienced a very remarkable and unpleasant sensation, which I referred chiefly to the epigastric region, but the exact nature of which I cannot describe, because I know nothing precisely similar to it. At the same time there was a sense of weakness in my limbs, as if I had not the command of my muscles, and I thought that I was about to fall. However, these sensations were momentary, and I experienced no inconvenience whatever afterwards.

I afterwards applied a more minute quantity of the essential oil to my tongue several times, without experiencing from it any disagreeable effects; but on applying a larger quantity, I was affected with the same momentary sensations as in the former instance, and there was a recurrence of them in three or four seconds after the first attack had subsided.

From the instantaneousness, with which the effects are produced; and from its acting more speedily when applied to the tongue, than when injected into the intestine, though the latter presents a better absorbing surface, we may conclude that this poison acts on the brain through the medium of the nerves, without being absorbed into the circulation.

### *Experiment with the Juice of the Leaves of Aconite.*

*Exp.* 7. An ounce of this juice was injected into the rectum of a cat. Three minutes afterwards he voided what appeared to be nearly the whole of the injection; he then stood for for some minutes perfectly motionless, with his legs drawn together; at the end of nine minutes, from the time of the injection, he retched and vomited; then attempted to walk, but faultered and fell at every step, as if from giddiness. At the end of thirteen minutes, he lay on one side insensible, motionless, except some slight convulsive motions of the limbs. The respiration became slow and laboured; and at forty-seven minutes from the time of the injection, he was apparently dead. One minute and a half afterwards, the heart was found contracting regularly one hundred times in a minute.

It appears from this experiment, that the juice of aconite, when injected into the intestine, occasions death by destroying the functions of the brain. From the analogy of other poisons, it is rendered probable that it acts on the brain through the medium of the nerves, without being absorbed into the circulation. This opinion is confirmed by the following circumstance: if a small quantity of the leaf of aconite is chewed, it occasions a remarkable sense of numbness of the lips and gums, which does not subside for two or three hours.

### *Experiments with the Infusion of Tobacco.*

*Exp.* 8. Four ounces of infusion of tobacco were injected into the rectum of a dog. Four minutes afterwards he retched, but did not vomit; he then became faint, and lay motionless on one side; at the end of nine minutes from the time of the injection, the heart could not be felt; he gasped for breath at long intervals; and in another minute there was no appearance whatever of life. I immediately laid open the cavities of the thorax and abdomen. The heart was much distended, and had entirely ceased to contract; there was no peristaltic motion of the intestines.

*Exp.* 9. An ounce of very strong infusion of tobacco was injected into the rectum of a cat. Symptoms were produced similar to those which occurred in the last experiment, and the animal died at the end of seven minutes from the time of the injection. On opening the thorax immediately after death, the heart was found extremely distended, and to have entirely ceased acting, with the exception of a slight tremulous motion of the auricles.

*Exp.* 10. Three ounces of infusion of tobacco were injected into the rectum of a dog. He was affected with symptoms similar to those in the former experiments, and died at the end of ten minutes. On opening the thorax immediately after death, I found the heart much distended, and to have entirely ceased contracting.

*Exp.* 11.

*Exp.* 11. Three ounces of infusion of tobacco were injected into the rectum of a dog. Immediately there took place tremulous contractions of the voluntary muscles. Five minutes afterwards the injection was repeated in the same quantity. The dog then was sick, and threw up some of the infusion with other matter from the stomach; he became faint, and died ten minutes after the second injection. Immediately after respiration had ceased I opened the thorax and found the heart extremely distended, and without any evident contraction, except of the appendix of the right auricle, which every now and then contracted in a slight degree. I divided the pericardium on the right side. In consequence of the extreme distension of the heart, this could not be done without irritating the fibres with the point of the scalpel. Immediately both auricles and ventricles began to contract with considerable force, so as to restore the circulation. Artificial respiration was produced, and the circulation was kept up for more than half an hour, beyond which time the experiment was not continued.

We may conclude from these experiments, that the effect of the infusion of tobacco, when injected into the intestine of a living animal, is to destroy the action of the heart, stopping the circulation and producing syncope. It appeared to me that the action of the heart ceased even before the animal had ceased to respire; and this was confirmed by another experiment in which, in a dog killed by the infusion of tobacco, I found the cavities of the left side of the heart to contain scarlet blood, while in those of the right side the blood was dark coloured. This poison therefore differs materially from alcohol, the essential oil of almonds, and the juice of aconite, which have no direct influence on the action of the heart. The infusion of tobacco renders the heart insensible to the stimulus of the blood, but it does not altogether destroy the power of muscular contraction, since the heart resumed its action in one instance on the division of the pericardium, and I have found that the voluntary muscles of an animal killed by this poison, are as readily stimulated to contract by the influence of the Voltaic battery, as if it had been killed in any other manner. At the same time, however, that the infusion of tobacco destroys the action of the heart, it appears to destroy also the functions of the brain, since these did not return in the last experiment; although the circulation was restored and kept up by artificial respiration.

Since there is no direct communication between the intestinal canal and the heart, I was at first induced to suppose that the latter becomes affected in consequence of the infusion being conveyed into the blood by absorption. Some circumstances cumstances in the following experiment have since led me to doubt whether this is the case.

*Exp.* 12. In a dog, whose head was removed, I kept up the circulation by means of artificial respiration, in the manner already described in the account of some experiments which I lately communicated to this Society. I then injected into the stomach and intestines, nine ounces of infusion of tobacco. At the time of the injection, the body of the animal lay perfectly quiet and motionless on the table; the heart acted regularly one hundred times in a minute. Ten minutes afterwards the pulse rose to one hundred and forty in a minute; the peristaltic motion of the intestines was much increased, and the voluntary muscles in every part of the body were thrown into repeated and violent spasmodic action. The joints of the extremities were alternately bent and extended, the muscles of the spine, abdomen, and tail alternately relaxed and contracted, so as to turn the whole animal from one side to the other. I have observed, in other instances, spasmodic actions of the muscles, where the circulation was kept up by artificial respiration, after the removal of the head, but not at all to be compared, either in strength or frequency, with those which took place on this occasion. I made pressure on the abdominal aorta for more than a minute, so as to obstruct the circulation of the blood in the lower extremities; but the muscular contractions were not lessened in consequence. Half an hour after the injection of the infusion the artificial respiration was discontinued. The heart continued to act, circulating dark coloured blood; the muscular contractions continued, but gradually diminishsd in strength and frequency. I tied a ligature round the vessels at the base of the heart, so as to stop the circulation, nevertheless the muscular contraction still continued, though less frequent and forcible than before, and some minutes elapsed before they entirely ceased.

In this experiment the disposition to contraction in the muscles was very much increased, instead of being diminished as in those just related. If the infusion of tobacco influences the heart from being absorbed into the blood, and thus coming into actual contact with its fibres, there is no evident reason why the removal of the brain and the employment of artificial respiration, should occasion so material a difference in its effects. If the contraction of the voluntary muscles had depended on the infusion circulating with the blood, it is reasonable to suppose that the pressure on the aorta would have occasioned some diminution of them, and that the complete obstruction of the circulation would have caused them to cease altogether.

From

From these considerations, I am induced, on the whole, to believe, that the infusion of tobacco when injected into the intestines, influences the heart through the medium of the nervous system: but I have not been able to devise any experiment, by which the truth or fallacy of this opinion might be put beyond the reach of doubt.

It appears remarkable, that the brain and nervous system, although not necessary to the action of the heart, should, when under the influence of the infusion of tobacco, be capable of influencing this organ so as to stop its action: but this is analogous to what we see occur in consequence of violent emotions of the mind. Those states of the nervous system, which accompany the passions of joy, fear, and anger, when existing in a moderate degree, render the heart more sensible to the stimulus of the blood, and increase the frequency of its contractions; while, when the same passions exist in a greater degree, the heart is rendered altogether insensible to the stimulus of the blood, and syncope ensues.

### *Experiments with the Empyreumatic Oil of Tobacco**.

*Exp.* 13. Less than a drop of this oil was applied to the tongue of a young cat. Instantly violent convulsions took place in all the muscles, and the respirations became very frequent. In five minutes after the application, she lay on one side insensible, with slight spasmodic actions of the muscles. At the end of eleven minutes, she retched but did not vomit. In a quarter of an hour she appeared to be recovering. I repeated the application of the poison, and she was again seized with violent convulsions, and became insensible, breathing at long intervals, and in two minutes from the second application respiration had entirely ceased, and she was apparently dead. On opening the thorax, I found the heart acting with regularity and strength, circulating dark-coloured blood. I introduced a tube into the trachea, and produced artificial respiration; the contractions of the heart became augmented in force and frequency, and there was no evident diminution in six or seven minutes, during which the artificial respiration was continued.

On dissection, nothing remarkable was found in the appearance of the tongue or brain.

---

* I was furnished with the empyreumatic oil of tobacco by Mr. W. Brande. It may be produced by subjecting the leaves of tobacco to distillation in a heat above that of boiling water: a quantity of watery fluid comes over, on the surface of which is a thin film of unctuous substance.

The symptoms and mode of death in this experiment did not essentially differ from those produced by the essential oil of almonds. I was surprised to find the effects of the empyreumatic oil so entirely different from those of the infusion of tobacco. Supposing that this difference might arise from the poison being more concentrated in the oil than in the infusion, I made the following experiments.

*Exp.* 14. A drop of the oil of tobacco was suspended in an ounce and a half of water by means of mucilage of gum arabic, and the whole was injected into the rectum of a dog. In two minutes afterwards he became faint, retched, but did not vomit. He appeared to be recovering from this state, and in twenty-five minutes after the first injection, it was repeated in the same quantity. He was then seized with symptoms similar to those in the last experiment, and in two minutes and a half he was apparently dead.

Two minutes after apparent death, on the thorax being opened into, the heart was found acting regularly one hundred times in a minute, and it continued acting for several minutes.

*Exp.* 15. A drop of the empyreumatic oil of tobacco with an ounce of water was injected into the rectum of a cat. The symptoms produced were, in essential circumstances, similar to those which occurred in the last experiment. The animal was apparently dead in five minutes after the injection, and the heart continued to contract for several minutes afterwards.

We may conclude from these experiments, that the empyreumatic oil of tobacco, whether applied to the tongue, or injected into the intestine, does not stop the action of the heart and induce syncope, like the infusion of tobacco; but that it occasions death by destroying the functions of the brain, without directly acting on the circulation. In other words, its effects are similar to those of alcohol, the juice of aconite, and the essential oil of almonds.

### III. *Experiments with Poisons applied to wounded Surfaces.*

#### *Experiments with the Essential Oil of Almonds.*

*Exp.* 16. I made an incision in the thigh of a rabbit, and introduced two drops of essential oil between the skin and the muscles. In four minutes after the application, he was seized with violent convulsions, and became insensible, and in two minutes more he was apparently dead; but the heart was felt through the ribs acting one hundred and twenty times in a minute, and it continued acting for several minutes.

There

There were no other appearances in the limb, than would have resulted from an ordinary wound.

*Exp.* 17. Two drops of the essential oil of almonds were introduced into a wound in the side of a mouse. Two minutes afterwards he was affected with symptoms similar to those which occurred in the last experiment, and in two minutes more he was apparently dead, but the heart continued to contract for some minutes afterwards.

From the experiments which I have just related, and from others which it appears unnecessary to detail, as the general results were the same, I have learned that where the essential oil of almonds is applied to a wound, its effects are not so instantaneous as when it is applied to the tongue; otherwise there is no difference in its effects in whatever manner it is applied.

### *Experiments with the Juice of the Leaves of the Aconite.*

*Exp.* 18. I made a wound in the side of a young rabbit, and introduced, between the skin and muscles, about twenty drops of the juice of aconite. Twenty-three minutes afterwards he was affected with symptoms, in all essential respects, similar to those, which occurred in an experiment already related, where the juice was injected into the rectum, and at the end of forty-seven minutes from the application of the poison, he was apparently dead. Two minutes after apparent death, the heart was found contracting, but very feebly.

### *Experiments with the Woorara*.*

*Exp.* 19. A small quantity of the woorara in powder was applied to a wound in the side of a Guinea pig. In ten minutes afterwards the animal was unable to walk; then he became quite motionless, except some slight occasional convulsions. He gradually became insensible, the respirations were laboured, and at the end of fourteen minutes from the application of the poison, the respiration had entirely ceased, and he was apparently dead; but on opening the thorax, the heart was found acting seventy times in a minutes, circulating dark coloured blood, and it continued to contract for several

* The Woorara is a poison with which the Indians of Guiana arm the points of their arrows. It appears not to differ essentially from the Ticunas, which was employed in the experiments of the Abbé Fontana. I am indebted to Dr. E. N. Bancroft, who not only furnished me with some of the Woorara which he had in his possession, but also lent me his assistance in the experiments which were made with it.

minutes afterwards. On dissection no preternatural appearances were observed in the brain; nor was there any other appearance in the limb than would have arisen from an ordinary wound.

*Exp.* 20. I made a wound in the side of a Guinea pig, and introduced into it about two grains of the woorara in powder. At the end of twenty-five minutes symptoms took place very similar to those which occurred in the last experiment, and in thirteen minutes more the animal was apparently dead; but the heart continued to contract one hundred and eight times in a minute, and by means of artificial respiration the circulation was kept up for more than twenty minutes.

The results of other experiments which I have made with the woorara, were similar to those just described. The heart continued to act after apparent death, and the circulation might be kept up by means of artificial respiration. It is evident that this poison acts in some way or another on the brain, and that the cessation of the functions of this organ is the immediate cause of death.

I found in these experiments, that the best mode of applying the woorara, is when it is disolved in water to the consistence of a thin paste. I first made the wound, and then smeared the poison over it with the end of the scalpel. I found that the animal was more speedily and certainly affected, if there was no hæmorrhage, unless the hæmorrhage was very copious, when it produced an opposite effect, by washing the poison away from the wound. When the poison was applied in large quantity, it sometimes began to act in six or seven minutes. Never more than half an hour elapsed from the time of the poison being inserted, to that of the animal being affected, except in one instance where a ligature was applied on the limb, which will be mentioned afterwards. The woorara which I employed had been preserved for some years, which will account for its having been less active than it has been described to be, by those who had witnessed its effects when in a recent state.

### *Experiments with the Upas Antiar*.*

*Exp.* 21. About two grains of this poison were made into a thin paste with water, and inserted into a wound in the thigh

* We are informed that the island of Java produces two powerful vegetable poisons, to one of which the natives give the name of *Upas tieuté*, and to the other that of *Upas antiar*. I was supplied with a quantity of the latter through the kindness of Mr. Marsden, who had some of it in his possession.

thigh of a dog. Twelve minutes afterwards he became languid; at the end of fifteen minutes, the heart was found to beat very irregularly, and with frequent intermissions; after this, he had a slight rigor. At the end of twenty minutes the heart beat very feebly and irregularly; he was languid; was sick and vomited; but the respirations were as frequent and as full as under natural circumstances, and he was perfectly sensible. At the end of twenty minutes he suddenly fell on one side, and was apparently dead. I immediately opened into the thorax, and found the heart distended with blood in a very remarkable degree, and to have entirely ceased contracting. There was one distinct and full inspiration after I had begun making the incision into the thorax. The cavities of the left side of the heart contained scarlet blood, and those of the right side contained dark coloured blood, as in a living animal.

*Exp.* 22. A small quantity of the upas antiar, prepared as before, was inserted into a wound in the thigh of a young cat. She appeared languid in two minutes after the poison was inserted. The symptoms which took place did not essentially differ from those which occurred in the last experiment, except that there were some convulsive motions of the limbs. At eight minutes after the poison was inserted she lay on one side, motionless and insensible, the heart could not be felt, but the respiration had not entirely ceased. On opening into the thorax, I found the heart to have ceased contracting. It was much distended with blood: and the blood in the cavities of the left side was of a scarlet colour. There were two full inspirations after the incision of the thorax was begun. On irritating the heart with the point of the scalpel, slight contractions took place in the fibres of the appendices of the auricles, but none in any other part.

*Exp.* 23. The experiment was repeated on a rabbit. The symptoms produced were similar to those in the last experiment; but the animal did not vomit, and the convulsive motions were in a less degree: he died eleven minutes after the poison was inserted. On opening the chest, the heart was found to have entirely ceased contracting; it was much distended with blood; and the blood in the cavities of the left side was of a scarlet colour. On irritating the heart with the point of the scalpel, the ventricles contracted, but not sufficiently to restore the circulation.

*Exp.* 24. About a grain of the upas antiar was inserted into a wound in the side of a rabbit. He was affected with symptoms similar to those before described, and died in ten minutes after the poison was applied. On opening the thorax immediately after death, the heart was found to have ceased ceased contracting, and the blood in the cavities of the left side was of a scarlet colour.

It appears from these experiments, that the upas antiar, when inserted into a wound, produces death (as infusion of tobacco does when injected into the intestine) by rendering the heart insensible to the stimulus of the blood, and stopping the circulation. The heart beats feebly and irregularly before either the functions of the mind, or the respiration appear to be affected. Respiration is performed even after the circulation has ceased; and the left side of the heart is found after death to contain scarlet blood, which never can be the case, where the cause of death is the cessation of the functions of the brain or lungs. The convulsions, which occur when the circulation has nearly ceased, probably arise from the diminution of the supply of blood to the brain, resembling those, which take place in a person, who is dying from hæmorrhage.

There remains an interesting subject of inquiry, "through what medium do poisons influence the brain when applied to wounds?" That poisons applied in this manner do not produce their effects precisely in the same way as poisons taken internally, is rendered probable by this circumstance; that some poisons, which are very powerful when applied to wounds even in small quantities, are either altogether inefficient when taken internally, or require to be given in very large quantities, in order to produce their effect, and vice versa.

A poison applied to a wounded surface may be supposed to act on the brain in one of three ways,

1. By means of the nerves, like poisons taken internally.

2. By passing into the circulation through the absorbent vessels.

3. By passing directly into the circulation through the divided veins.

*Exp.* 25. In order to ascertain whether the woorara acts through the medium of the nerves, I exposed the axilla of a rabbit, and divided the spinal nerves supplying the upper extremity, just before they unite to form the axillary plexus. The operation was performed with the greatest care. I not only divided every nervous filament, however small, which I could detect, but every portion of cellular membrane in the axilla, so that the artery and vein were left entirely insulated. I then made two wounds in the fore-arm, and inserted into them some of the woorara formed into a paste. Fourteen minutes after the poison was applied, the hind legs became paralytic, and in ten minutes more he died, with symptoms precisely similar to those which took place in the former experiments, periments, and the heart continued to act after apparent death. On dissection, the nerves of the upper extremity were particularly examined, but not the smallest filament could be found undivided.

I made the following experiment to ascertain whether the woorara passes into the circulation through the absorbent vessels.

*Exp.* 26. I tied a ligature round the thoracic duct of a dog, just before it perforates the angle of the left subclavian and jugular veins. I then made two wounds in the left hind leg, and introduced some of the woorara in powder into them. In less than a quarter of an hour he became affected with the usual symptoms, and died in a few minutes afterwards.

After death, I dissected the thoracic duct with great care. I found it to have been perfectly secured by the ligature. It was very much distended with chyle, and about two inches below its termination its coats had given way, and chyle was extravasated into the cellular membrane. The lymphatic vessels in the left axilla were distended in a very remarkable degree, and on dividing them, not less than a dram of lymph issued from the divided ends.

Since neither the division of the nerves, nor the obstruction of the thoracic duct interfere in the slightest degree with the effects of the woorara, there is presumptive evidence that it acts on the brain by entering the circulation through the divided veins. I endeavoured to ascertain, by experiment, whether this is really the case.

To apply ligatures to the large vessels of a limb only would evidently lead to no satisfactory conclusion, since the anastomosing vessels might still carry on the circulation. The only way, which I could devise of performing the experiment, was to include all the vessels, small as well as large, in a ligature.

*Exp.* 27. In order to make the experiment more satisfactorily, I exposed the sciatic nerve of a rabbit in the upper and posterior part of the thigh, and passed under it a tape half an inch wide. I then made a wound in the leg, and having introduced into it some of the woorara mixed with water, I tied the tape moderately tight on the fore-part of the thigh. Thus I interrupted the communication between the wounds and the other parts of the body, by means of the vessels, while that by means of the nerve still remained. After the ligature was tightened, I applied the woorara a second time, in another part of the leg. The rabbit was not at all affected, and at the end of an hour I removed the ligature. Being engaged in some other pursuit, I did not watch the animal so closely as I should otherwise have done; but twenty minutes after the ligature ligature was removed, I found him lying on one side, motionless and insensible, evidently under the influence of the poison, but the symptoms were less violent than in most instances, and after lying in this state he recovered, and the limb became perfectly warm, and he regained the power of using it.

*Exp.* 28. I repeated the last experiment with this difference, that after having applied the poison, I made the ligature as tight as I could draw it. I removed the ligature at the end of an hour and twenty minutes, but the animal was not at all affected either before or after the removal of the ligature, and on the following day he had recovered the use of the limb.

*Exp.* 29. I repeated the experiment a third time, drawing the ligature very tight. At the end of forty-five minutes, the animal continued perfectly well, and the ligature was removed. I watched him for three quarters of an hour afterwards, but there were no symptoms of his being affected by the poison. On the following day the rabbit died, but this I attribute to the injury done to the limb and sciatic nerve by the ligature, as there was the appearance of inflammation in the parts in the neighbourhood of the ligature.

These three experiments were made with the greatest care. From the mode in which the poison was applied, from the quantity employed, and from my prior experience, I should have entertained not the smallest doubt of the poison taking effect in every instance in less than twenty minutes, if no ligature had been applied. In two of the three, the quantity of woorara was more than had been used in any former experiments.

I have not judged it necessary to make any more experiments, with the ligature on the limb, because the numerous experiments of the Abbé Fontana on the ticunas coincide in their results with those which have just been detailed, and fully establish the efficacy of the ligature, in preventing the action of the poison. It is not to be wondered at, that the ligature should sometimes fail in its effects, since these must evidently depend on the degree in which the circulation is obstructed, and on the length of time during which the obstruction is continued.

There can be little doubt that the woorara affects the brain, by passing into the circulation through the divided vessels. It is probable that it does not produce its effects, until it enters the substance of the brain, along with the blood, in which it is dissolved; nor will the experiments of the Abbé Fontana, in which he found the ticunas produce almost instant death when injected into the jugular vein of a rabbit, be found to militate against this conclusion, when we consider how short is the distance, which, in so small an animal, the blood has to pass from the jugular vein to the carotid artery, and the great rapidity of the circulation; since in a rabbit under the influence of terror, during such an experiment, the heart cannot be supposed to act so seldom as three times in a second.

I have made no experiments to ascertain through what medium other poisons when applied to wounds affect the vital organs, but from analogy we may suppose that they enter the circulation through the divided blood-vessels.

### IV.

The facts already related led me to conclude that alcohol, the essential oil of almonds, the juice of aconite, the oil of tobacco, and the woorara, occasion death simply by destroying the functions of the brain. The following experiment appears fully to establish the truth of this conclusion.

*Exp.* 30. The temperature of the room being 58° of Fahrenheit's thermometer, I made two wounds in the side of a rabbit, and applied to them some of the woorara in the form of paste. In seven minutes after the application, the hind legs were paralysed, and in fifteen minutes respiration had ceased, and he was apparently dead. Two minutes afterwards the heart was still beating, and a tube was introduced through an opening into the trachea, by means of which the lungs were inflated. The artificial respiration was made regularly about thirty-six times in a minute.

At first, the heart contracted one hundred times in a minute.

At the end of forty minutes, the pulse had risen to one hundred and twenty in a minute.

At the end of an hour, it had risen to one hundred and forty in a minute.

At the end of an hour and twenty-three minutes, the pulse had fallen to a hundred, and the artificial respiration was discontinued.

At the commencement of the experiment, the ball of a thermometer being placed in the rectum, the quicksilver rose to one hundred degrees; at the close of the experiment it had fallen to eighty-eight and a half.

During the continuance of the artificial respiration, the blood in the femoral artery was of a florid red, and that in the femoral vein of a dark colour, as usual.

It has been observed by M. Bichat, that the immediate cause of death, when it takes place suddenly, must be the cessation of the functions of the heart, the brain, or the lungs. This observation may be extended to death under all circumstances. The stomach, the liver, the kidneys, and many other organs are necessary to life, but their constant action is not necessary; and the cessation of their functions cannot therefore be the *immediate* cause of death. As in this case the action of the heart had never ceased; as the circulation of the blood was kept up by artificial respiration for more than an hour and twenty minutes after the poison had produced its full effects; and as during this time the usual changes in the colour of the blood took place in the lungs; it is evident that the functions of the heart and lungs were unimpaired: but that those of the brain had ceased, is proved, by the animal having continued in a state of complete insensibility, and by this circumstance, that animal heat, to the generation of which I have formerly shewn the influence of the brain to be necessary, was not generated.

Having learned that the circulation might be kept up by artificial respiration for a considerable time after the woorara had produced its full effects, it occurred to me that in an animal under the influence of this or of any other poison that acts in a similar manner, by continuing the artificial respiration for a sufficient length of time after natural respiration had ceased, the brain might recover from the impression, which the poison had produced, and the animal might be restored to life. In the last experiment, the animal gave no sign of returning sensibility; but it is to be observed, 1. That the quantity of the poison employed was very large. 2. That there was a great loss of animal heat, in consequence of the temperature of the room being much below the natural temperature of the animal, which could not therefore be considered under such favourable circumstances as to recovery, as if it had been kept in a higher temperature. 3. That the circulation was still vigorous when I left off inflating the lungs, and therefore it cannot be known what would have been the result, if the artificial respiration had been longer continued.

*Exp.* 30. A wound was made in the side of a rabbit, and one drop of the essential oil of almonds was inserted into it, and immediately the animal was placed in a temperature of 90°. In two minutes he was under the influence of the poison. The usual symptoms took place, and in three minutes more respiration had ceased, and he lay apparently dead, but the heart was still felt beating through the ribs. A tube was then introduced into one of the nostrils, and the lungs were inflated about thirty-five times in a minute. Six minutes after the commencement of artificial respiration, he moved his head and legs, and made an effort to breathe. He then was seized with convulsions, and again lay motionless, but continued to make occasional efforts to breathe. Sixteen minutes after its its commencement, the artificial respiration was discontinued. He now breathed spontaneously seventy times in a minute, and moved his head and extremities. After this, he occasionally rose, and attempted to walk. In the intervals, he continued in a dozing state; but from this he gradually recovered. In less than two hours he appeared perfectly well, and he continued well on the following day.

The inflating the lungs has been frequently recommended in cases of suffocation, where the cause of death is the cessation of the functions of the lungs: as far as I know, it has not been before proposed in those cases, in which the cause of death is the cessation of the functions of the brain.* It is probable that this method of treatment might be employed with advantage for the recovery of persons labouring under the effects of opium, and many other poisons.

V.

The experiments which have been detailed lead to the following conclusions.

1. Alcohol, the essential oil of almonds, the juice of aconite, the empyreumatic oil of tobacco, and the woorara, act as poisons by simply destroying the functions of the brain; universal death taking place, because respiration is under the influence of the brain, and ceases when its functions are destroyed.

2. The infusion of tobacco when injected into the intestine, and the upas antiar when applied to a wound, have the power of rendering the heart insensible to the stimulus of the blood, thus stopping the circulation; in other words, they occasion syncope.

3. There is reason to believe that the poisons, which in these experiments were applied internally, produce their effects through the medium of the nerves without being absorbed into the circulation.

4. When the woorara is applied to a wound, it produces its effects on the brain, by entering the circulation through the divided blood-vessels, and from analogy, we may con-

* Since this paper was read, I have been favoured by the Right Hon. the President with the perusal of a Dissertation on the Effects of the Upas Tieutè, lately published at Paris by M. Delile, by which I find that he had employed artificial respiration for the purpose of recovering animals, which were under the influence of this poison, with success. M. Delile describes the Upas Tieutè as causing death, by occasioning repeated and long continued contractions of the muscles of respiration, on which it acts through the medium of the spinal marrow, without destroying the functions of the brain.

 clude clude that other poisons, when applied to wounds, operate in a similar manner.

5. When an animal is apparently dead from the influence of a poison, which acts by simply destroying the functions of the brain, it may, in some instances, at least, be made to recover, if respiration is artificially produced, and continued for a certain length of time.

From analogy we might draw some conclusions respecting the mode in which some other vegetable poisons produce their effects on the animal system; but I forbear to enter into any speculative inquiries; as it is my wish, in the present communication, to record such facts only, as appear to be established by actual experiment.

